# Effect of a sport-for-health intervention (SmokeFree Sports) on smoking-related intentions and cognitions among 9-10 year old primary school children: a controlled trial

**DOI:** 10.1186/s12889-016-3048-3

**Published:** 2016-05-26

**Authors:** Ciara E. McGee, Joanne Trigwell, Stuart J. Fairclough, Rebecca C. Murphy, Lorna Porcellato, Michael Ussher, Lawrence Foweather

**Affiliations:** Centre for Public Health, Liverpool John Moores University, Henry Cotton Campus, 15-21 Webster Street, Liverpool, L3 2AT UK; Centre for Health Promotion Research, Leeds Beckett University, Calverley Building, City Campus, Leeds, LS1 3HE UK; Department of Sport and Physical Activity, Edge Hill University, St. Helens Road, Ormskirk, Lancashire L39 4QP UK; Physical Activity Exchange, Research Institute for Sport and Exercise Sciences, Liverpool John Moores University, 62 Great Crosshall Street, Liverpool, L3 2AT UK; Population Health Research Institute, St George’s, University of London, Cranmer Terrace, London, SW17 0RE UK; Department of Physical Education and Sport Sciences, University of Limerick, Limerick, Ireland

**Keywords:** Smoking, Prevention, Children, Intervention, School-based

## Abstract

**Background:**

Preventing children from smoking is a public health priority. This study evaluated the effects of a sport-for-health smoking prevention programme (SmokeFree Sports) on smoking-related intentions and cognitions among primary school children from deprived communities.

**Methods:**

A non-randomised-controlled trial targeted 9-10 year old children from Merseyside, North-West England. 32 primary schools received a programme of sport-for-health activities over 7 months; 11 comparison schools followed usual routines. Data were collected pre-intervention (T0), and at 8 months (T1) and one year post-intervention (T2). Smoking-related intentions and cognitions were assessed using an online questionnaire. Intervention effects were analysed using multi-level modelling (school, student), adjusted for baseline values and potential confounders. Mixed-sex focus groups (*n* = 18) were conducted at T1.

**Results:**

961 children completed all assessments and were included in the final analyses. There were no significant differences between the two study groups for non-smoking intentions (T1: β = 0.02, 95 % CI = -0.08–0.12; T2: β = 0.08, 95 % CI = -0.02–0.17) or for cigarette refusal self-efficacy (T1: β = 0.28, 95 % CI = -0.11–0.67; T2: β = 0.23, 95 % CI = -0.07–0.52). At T1 there was a positive intervention effect for cigarette refusal self-efficacy in girls (β = 0.72, 95 % CI = 0.21–1.23). Intervention participants were more likely to ‘definitely’ believe that: ‘it is not safe to smoke for a year or two as long as you quit after that’ (RR = 1.19, 95 % CI = 1.07–1.33), ‘it is difficult to quit smoking once started’ (RR = 1.56, 95 % CI = 1.38–1.76), ‘smoke from other peoples’ cigarettes is harmful’ (RR = 1.19, 95 % CI = 1.20–2.08), ‘smoking affects sports performance’ (RR = 1.73, 95 % CI = 1.59–1.88) and ‘smoking makes ‘no difference’ to weight’ (RR = 2.13, 95 % CI = 1.86–2.44). At T2, significant between-group differences remained just for ‘smoking affects sports performance’ (RR = 1.57, 95 % CI = 1.43–1.72). Focus groups showed that SFS made children determined to remain smoke free and that the interactive activities aided children’s understanding of smoking harms.

**Conclusion:**

SFS demonstrated short-term positive effects on smoking attitudes among children, and cigarette refusal self-efficacy among girls. Although no effects were observed for non-smoking intentions, children said that SFS made them more determined not to smoke. Most children had strong intentions not to smoke; therefore, smoking prevention programmes should perhaps target early adolescents, who are closer to the age of smoking onset.

## Background

Smoking is an addiction often initiated in childhood, with approximately 207,000 children taking up smoking each year in the UK [1]. The earlier a child starts to smoke, the higher their chances of becoming a regular smoker and the more difficult it becomes to quit [2]. Early onset of smoking and persisting in the habit in adulthood increases the risk of developing lung cancer and other smoking-related diseases [3, 4]. Preventing smoking uptake in children by de-normalising tobacco use is therefore a key public health priority for the UK Government [5], which is aiming for a tobacco-free generation by 2025 [6]. Although only 0.3 % of 8-10 year old UK primary school children have ever smoked [7], some children develop intentions to start smoking [8]. Behavioural intentions to smoke are worth paying special attention to as they are theorised to be the first step in smoking initiation [9]. Intentions to smoke may be explained by individual smoking-related cognitions such as attitudes towards smoking and cigarette refusal self-efficacy [10–12]. Since children living in socially deprived areas have a high intention to smoke [13] and harbour misconceptions about the harms of smoking [14], implementing a smoking prevention intervention in primary school may prevent children from starting to smoke.

Schools are considered an appropriate setting for smoking prevention because they can provide an efficient means of reaching large numbers of children [15] and provide an opportunity to ‘set’ healthy and enduring patterns of behaviour [16, 17]. Consequently, numerous school-based smoking prevention programmes have been developed and implemented over the last decade to discourage smoking uptake and deter regular use [18, 19]. Previous interventions have predominantly targeted adolescents, whilst programmes that have been developed for primary school aged children have been implemented outside the UK [18, 19]. Waiting until secondary school to intervene with smoking prevention programmes can be too late, since by then adolescents may have developed deep rooted smoking expectancies and norms [20–22] and, for some the behaviour is already underway (8 % of 15 year olds smoke) [23].

Sport-for-health programmes use sport as a mechanism to promote health and prevent disease [24–27]. Interventions typically use participatory approaches like game-based learning and activities with sport coaches, who represent important role models for youth, to transmit health promotion messages and positively shape attitudes [28, 29]. The use of sport as an educational platform for tobacco control has previously been trialled in the US and Canada with initiatives such as Tobacco Free Sports [30], Tobacco Free Athletes [31] and Play, Live, Be Tobacco Free [32]. Sport-for-health interventions have several potential benefits over traditional classroom-based smoking prevention education approaches. First, participation in physical activity improves health not only directly but also through its protective effect against smoking initiation in youth [33, 34]. Second, given that all primary school children are required to participate in physical education, this lesson could provide a forum to integrate smoking education messages within the primary school curriculum. Third, the use of sport as a smoking prevention strategy encourages active engagement with the intervention as well as interactions with other pupils and teaching staff, and is consistent with National Institute for Health and Care Excellence (NICE) recommendations [35] to deliver interactive and participatory smoking prevention interventions. Finally, integrating physical activity into the learning process may enable children to efficiently retain and retrieve learned information [28, 36–38]. To the authors’ knowledge, no published study has evaluated the use of sport-for-health programmes for smoking prevention in the UK.

SmokeFree Sports (SFS) was a sport-for-health smoking prevention intervention for youth in Liverpool, which is one of the most deprived communities in England [39] where addressing inequalities in tobacco use is a public health priority. Established in October 2010, SFS was commissioned as part of the ‘SmokeFree Liverpool’ public health campaign. The intervention was designed in accordance with the NICE guidance [35] and the Medical Research Council (MRC) framework for developing and evaluating complex interventions [40]. Phase one of SFS (February-June 2011) was a community feasibility trial in five youth clubs, which received 12 weeks of coaching activities (dance, dodge-ball and boxing) delivered by trained sports coaches [29, 41]. A formative evaluation demonstrated that the intervention helped to prevent youth from initiating smoking and had positive benefits on their attitudes and knowledge about smoking [29, 41]. However, coaches reported challenges associated with its delivery in youth clubs and recommended that the intervention be trialled in schools. Phase two (February-April 2012) therefore examined the feasibility of a six week intervention in three primary schools [42]. Trained coaches delivered twelve sessions of sports (football and dance). Similar positive benefits for children were observed, whilst teachers and coaches perceived SFS to be acceptable for smoking prevention education [42]. These promising results led to the development of a larger, controlled trial to investigate the effectiveness of SFS in Liverpool primary schools.

The present study evaluated whether SFS, a sport-for-health smoking prevention intervention, is effective in increasing non-smoking intentions in 9-10 year old primary school children from Liverpool, immediately post-intervention and at a follow-up one year later. Secondary aims were to investigate the impact of the intervention on children’s attitudes towards smoking and cigarette refusal self-efficacy, termed smoking-related cognitions hereafter. The study also investigated whether sex moderated the intervention effects as differences in cognitive vulnerability towards smoking have been found between preadolescent boys and girls [14]. In addition, focus groups with children were conducted to produce more complete knowledge to inform interpretations of intervention effectiveness. Since sport-for-health interventions are an emergent area of health promotion research where evaluations are sparse and/or have lacked methodological rigour [26, 43], it is recognised that rigorous evaluations of interventions are needed to inform future practice and procedures [25].

## Methods

### Study design

A school-based non-randomised controlled trial was conducted to evaluate the effect of a sport-for-health smoking prevention intervention, SmokeFree Sports on children’s intentions (not) to smoke and smoking-related cognitions. Due to funding requirements, SFS was offered to all primary schools within the Liverpool City Council administrative boundaries and therefore an a priori sample size calculation was not undertaken. Schools within Knowsley, another metropolitan borough in Merseyside with similar characteristics to Liverpool in terms of adult smoking rates (Liverpool: 24.2 %; Knowsley: 27.6 %) [44], deprivation levels [45] and ethnic composition [46], were recruited as comparison schools. For logistical reasons, it was not possible to blind the research team to the group-allocation. Schools were clustered into two groups:

1. Intervention group (Liverpool): Schools received their usual smoking-related education plus SFS
2. Comparison group (Knowsley): Schools received their usual smoking education only

**Fig. 1:**
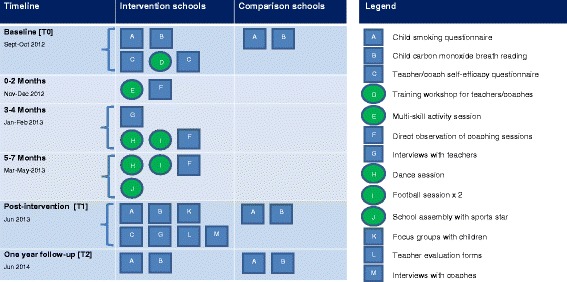
Schematic overview of SmokeFree Sports intervention and evaluation components

A schematic overview of the intervention and evaluation components is shown in Fig. 1. Data collection occurred over 20 months with measurements at baseline (T0, September and October 2012) and post-intervention (T1, June 2013) whilst children were in Year 5 of primary school, and at one year after the intervention had finished (T2, June 2014; Year 6 of primary school). Ethical approval for the study was granted by Liverpool John Moores University Research Ethics Committee (12/SPS/038).

### Participants and recruitment

In September 2012, all eligible primary schools (mainstream state schools; *n* = 154), from Liverpool (*n* = 104) and Knowsley (*n* = 50), were invited to participate in the study via email, post and telephone. Once schools had given written informed consent to participate in the study, all Year 5 children (aged 9–10 years; *n* = 1393) were invited to take part. Parents/guardians received a letter containing a parent and child information sheet and opt-out form. Parents/guardians were given contact information for the research team to discuss the project and could opt their child out of the study by telephone or by signing and returning the opt-out form. At data collection, children were given a verbal explanation of the study and asked to give signed assent. Children could withdraw from the research study at any time.

### Intervention

The SFS intervention was delivered during school hours in Liverpool primary schools between October 2012 and May 2013. The intervention focused on smoking prevention and therefore Year 5 children (aged 9-10) were identified as an important cohort to target. Further, NICE [35] guidelines postulate that smoking prevention efforts would be most effective if they began in primary school.

A detailed description of the SFS intervention has been published elsewhere [47, 48]. Briefly, the socio-ecological model [49] and cognitive theories including the Health Belief Model [50], Theory of Planned Behaviour [9] and Social Cognitive/Learning [51], guided the intervention design. The intervention aimed to implement a programme of smoking prevention actions through fun, participatory and interactive sports activities delivered by teachers and coaches. Intervention components included provision of professional development training, a training manual including 10 session plans, five coaching sessions delivered by SFS coaches, a school assembly with a local sports star, sports equipment as incentives for teachers to deliver and evaluate a minimum of five SFS sessions, a smoke free pledge for children to sign, and incentives for children for participating in the research (SFS water bottle, drawstring bag and pen). Behaviour change techniques [52] used with children included a behavioural contract (smoke free pledge); social support, advice, verbal persuasion and positive reinforcement (from peers, teachers and coaches) on remaining never smokers; information and salience of the social, emotional and health consequences of smoking; an exploration of the pros and cons of smoking; awareness raising of regret children will feel if they smoke; social comparisons with peers to dispel myths that smoking is normative; modelling of never smoking (from elite athletes); cigarette refusal rehearsals, and the promotion of physical activity as a positive distraction to smoking.

### Comparison group

Children in the comparison group were requested to follow their usual smoking education. It is not mandatory to address smoking education in Key Stage 2 (pupils aged 7–11) of the UK National Curriculum [53], and it is at schools’ discretion to include the topic as part of Personal Social Health and Economic education. On completion of data collection at one year follow-up, comparison schools received a copy of the SFS training manual, and children were given a SFS water bottle, drawstring bag and pen for participating in the research study.

### Measures

The primary outcome measure was intentions (not) to smoke of the participating children; secondary outcomes included individual smoking-related cognitions (attitudes and refusal self-efficacy). Outcomes were assessed based on a self-reported questionnaire completed by children at T0, T1 and T2, and through focus groups with children, which were conducted at T1 only due to funding restrictions.

#### Smoking questionnaire

A questionnaire was constructed using items adapted from questionnaires previously used with this age group [54–57]. Background demographic information has been described in detail elsewhere [14]. Briefly, demographics assessed included age (years), gender (1 = girl, 0 = boy), ethnicity (1 = white British, 0 = other) and SES (home postcodes were used to generate indices of multiple deprivation (IMD) scores [39]. Children’s enjoyment of physical activity was assessed using the 16 item Physical Activity Enjoyment Scale [58]. Parent, sibling and friend smoking behaviour (1 = smokes (parent/sibling/friend) or tried (friend only), 0 = non-smoker) was assessed using an item from the Health Survey for England [57]. Child smoking behaviour (0 = never smoked, 1 = ever smoked) was also measured for descriptive purposes using a single item from the Health Survey for England [57]. As an indicator of smoking status, expired carbon monoxide (CO) concentrations were taken in private and recorded using a piCOsimple Smokerlyzer (Bedfont Scientific UK, England) with a reading above 10 ppm used as cut-off for defining smokers [59].

Intention (not) to smoke was assessed using two items from the Health Survey for England [57], ‘Do you think you will smoke in the next month/year?’, as well as an item designed by the research team ‘Do you think you will smoke in secondary school?’. Responses ranged from ‘definitely yes’ (1) to ‘definitely not’ (4) and were summed to produce a total intention score (range 3–12). A high score on total intention indicated a strong intention not to smoke. Cronbach alpha for total intention showed good internal consistency (α = 0.81).

Refusal self-efficacy was measured using three items adapted from a nine-item self-efficacy scale in adolescents [54]. Items assessed the child’s confidence in their ability to be a non-smoker and refuse cigarettes in different situations. Responses consisted of Likert scales ranging from ‘not confident at all’ (1) to ‘very confident’ (5) and were summed to create a total refusal self-efficacy score (range 3–15). Cronbach alpha for the combined scale showed good internal consistency (α = 0.81). A high score on the scale indicated a high level of refusal self-efficacy.

Attitude structure includes affective, behavioural and cognitive components [60]. For the purpose of this study, the cognitive component of children’s attitudes was explored through items adapted from the Global Youth Tobacco Survey (GYTS) [56] and the Health Survey for England [57], including ‘Do you think smoking is bad for your health?’, ‘Once someone has started smoking, do you think it will be difficult to quit?’, ‘Do you think that it is safe to smoke for only a year or two as long as you quit after that?’, ‘Do you think the smoke from other people’s cigarettes is harmful to you?’. An additional item ‘Do you think smoking effects sport performance?’ was developed by the research team. Responses ranged from ‘definitely not’ (1) to ‘definitely yes’ (4). A summary scale was created but internal consistency was low (α = .49). Since the data for individual attitude items were positively skewed and distribution was not improved by statistical transformation, responses were collapsed into dichotomous variables for analyses: a definitive negative attitude towards smoking (i.e. ‘definitely yes’) was scored 1; the remaining response categories (i.e. ‘probably yes’, ‘probably not’ and ‘definitely not’) indicated a more favourable attitude towards smoking and thus were collapsed into a single group and scored 0. One attitude item (‘Do you think that it is safe to smoke for only a year or two as long as you quit after that?’) was reverse coded in order to maintain consistent scale direction for all items. An additional attitude item, ‘Do you think smoking makes you gain weight?’ was also included from the Health Survey for England [57]. Whilst it is recognised that smoking is associated with weight loss [61], smoking is widely discouraged by public health professionals for weight control. Therefore, a key message included within the curriculum was that regular physical activity and healthy eating, but not smoking, was important for maintenance of a healthy weight. Thus responses for this item were collapsed into a dichotomous variable for analysis with ‘no difference’ scored 1 and the remaining response categories (i.e., ‘lose weight’ or ‘gain weight’) grouped and scored 0.

#### Focus groups with children

Eighteen mixed-sex focus groups with children (*n* = 95; 45 % boys) were facilitated by trained researchers immediately following the intervention [T1]. Focus groups comprised of five to six children, lasted between 30 and 50 min and were audio recorded using a Dictaphone. Children’s perceptions of smoking, appropriateness of the intervention, and improvements for future implementation were explored. Photographs of SFS games were used to help children recall activity type [62, 63]. To aid the credibility of data, facilitators’ reflected interpretations back to children during the focus groups. The present study focuses on children’s perceptions surrounding the impact of SFS on intentions (not) to smoke and individual smoking-related cognitions, thus other findings are discussed in the process evaluation paper, which has been published elsewhere [64].

### Analyses

Participants with missing data at either post-intervention [T1] or follow-up [T2] were not considered in the analyses (i.e., a complete case analysis). To describe the demographic characteristics of children at baseline [T0] and differences concerning primary (smoking intentions) and secondary outcomes (attitudes towards smoking and refusal self-efficacy), general descriptive analyses were conducted. Independent t-tests and chi-square tests were used to assess whether the primary and secondary outcomes differed between the study groups at baseline, and to assess differences between those participants included and excluded from the final analysis. Multilevel linear and logistic regression analyses examined intervention effects on the primary and secondary outcomes. To account for the clustering effect among children being nested in schools, a two-level data structure was conducted. Children were defined as the first level unit of analysis and schools the second level unit of analysis [65]. Two analyses were conducted for each of the outcome variables to examine the intervention effects. The first analysis determined the difference between the intervention and comparison group adjusting for baseline value of the outcome measure (‘crude’ analysis). The second analysis determined this effect when the covariates were added to the model (‘adjusted’ analysis); these covariates included age, ethnicity, deprivation level, mother/father/sibling/friend smoking, intentions to smoke and individual smoking-related cognitions, since these variables may influence each other [9, 51]. Additionally, physical activity enjoyment was adjusted for in the analysis because we hypothesised that children who enjoy physical activity may be more amenable to a sport-for-health intervention. In addition, separate analyses for boys and girls were performed to assess intervention effects between baseline and post-intervention, and baseline and one year follow-up. To determine whether the intervention effect was different for boys and girls, a dichotomous interaction term (labelled ‘sex’) was constructed. Regression coefficients in each model were assessed for significance using the Wald statistic with one degree of freedom. As the prevalence of negative attitudes towards smoking was high in both study arms, odds ratios were converted to relative risks [66] to avoid overestimation of effects and for ease of interpretation of results. Analyses were performed using IBM SPSS Statistics v.22 and MLwiN 2.30 software (Centre for Multi-level Modelling, University of Bristol, UK). Statistical significance was set at *p* < 0.05, and at *p* < 0.10 for the sex interaction term [66].

Child focus groups were transcribed verbatim, imported into NVivo 10 software, and subjected to thematic analysis [67]. This process involved reading and re-reading text and assigning broad thematic codes, some of which were pre-defined from topics covered in the group schedule. Subsequently, broad codes were collapsed into higher and lower order themes and descriptive and interpretive summaries were written based on recursive engagement with the data. A combination of inductive analysis and deductive techniques were used to generate codes. To aid the credibility and trustworthiness of the results, analyses and interpretations of the data were discussed amongst three members (CM, JT and LF) of the research team [67].

## Results

**Fig. 2:**
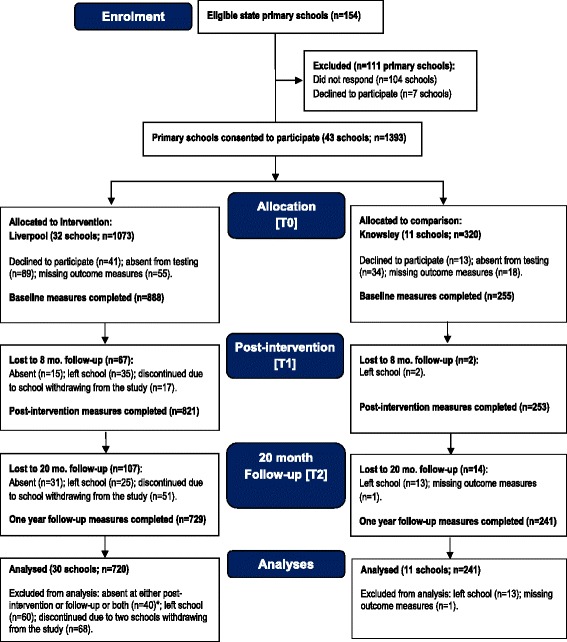
Flow of schools and participants through the study. *Six children were absent at both post-intervention and follow-up

Figure 2 shows the flow of schools and participants through the trial. In total, 43 schools participated in the study (27.9 % response rate), including 32 (31 %) from Liverpool and 11 (22 %) from Knowsley. Schools that declined to participate provided diverse reasons for not taking part (e.g., too busy, key teacher on sick leave, already in receipt of external projects). Of the 1393 potentially eligible children at T0, 1143 completed baseline measures (92 % response rate); 961 children completed assessments at T0, T1, and T2 and were included in the final analyses (84 % participation rate). Participant retention ranged from 80 % (T0) to 79 % (T2) in the comparison group. The intervention group’s retention ranged from 83 % at baseline to 68 % at T2. However, the withdrawal of two intervention schools due to internal staffing issues excluded 68 children. Had the schools not withdrawn, assuming all children would have continued through the study, the retention at follow-up would have been 74 %. Compared with intervention children included in the analyses, a higher proportion of intervention children that were excluded from the analyses had a sibling that smoked (*p* < 0.01) and a lower proportion believed that smoking is bad for health (*p* < 0.05). Other baseline values did not differ between those included and excluded.

Baseline characteristics for the final sample of child participants (mean age 9.6 ± 0.3 years, 50.4 % female, 98.3 % White British) are shown in Table 1. Over seven in ten (71.9 %) participating children lived within areas ranked within the highest 10 % for deprivation in England. The majority of children reported to have never smoked at T0 (97.5 %: comparison group, 96.3 %; intervention group, 97.9 %). CO readings were recorded for 82 % of participants and confirmed self-reported non-smoking status with all readings below 10 ppm (Mean = 1.3 ± 0.7 ppm). Over half of children (57.3 %) reported that at least one family member was a current smoker, whilst almost one in five children reported having a friend who smoked. Children generally had strong non-smoking intentions and high refusal self-efficacy expectations, though 40-58 % of children displayed more favourable attitudes towards smoking on five of the six attitude items. Children in the intervention group were less likely to be White British and were significantly more deprived (*p* < 0.01) than children in the comparison group. A higher proportion of intervention children, in particular girls, definitely believed that smoking was difficult to quit (*p* < 0.01) and that smoking affects sports performance (*p* < 0.05). No other significant group differences were found at T0.

**Table 1:** Sample characteristics of children at baseline

	Comparison			Intervention		
	All (*n* = 241) M ± SD or %	Boys (*n* = 114) M ± SD or %	Girls (*n* = 127) M ± SD or %	All (*n* = 720) M ± SD or %	Boys (*n* = 363) M ± SD or %	Girls (*n* = 357) M ± SD or %
Demographics						
Age (years)	9.6 ± 0.3	9.6 ± 0.3	9.6 ± 0.3	9.6 ± 0.3	9.6 ± 0.3	9.6 ± 0.3
Ethnicity (White British)	98.3	99.1	97.6	82.1	82.6	81.5
Deprivation level (IMD)	50.9 ± 17.9	49.8 ± 17.7	51.9 ± 18.2	55.7 ± 16.4	55.5 ± 16.5	55.8 ± 16.3
Enjoyment of physical activity						
PACES enjoyment scale (range 1–5)	4.3 ± 0.7	4.3 ± 0.7	4.3 ± 0.7	4.3 ± 0.7	4.2 ± 0.8	4.4 ± 0.6
Smoking intentions						
Total non-smoking intentions (range 4–12)	11.7 ± 1.0	11.6 ± 1.1	11.7 ± 0.9	11.8 ± 0.8	11.7 ± 1.0	11.9 ± 0.5
Self-efficacy						
Total refusal self-efficacy (range 3–15)	13.5 ± 3.2	13.5 ± 3.1	13.5 ± 3.3	13.7 ± 3.0	13.4 ± 3.3	14.0 ± 2.7
Attitudes towards smoking						
Smoking is bad for health (‘*definitely yes’*)	88.8	87.7	89.8	90.1	86.2	94.1
Safe to smoke year or two (‘*definitely not’*)	59.8	60.5	59.1	64.4	63.6	65.3
Difficult to quit once started (‘*definitely yes’*)	43.2	45.6	40.9	52.8	51.2	54.3
Others smoke harmful to you (‘*definitely yes’*)	59.3	60.5	58.3	66.1	63.1	69.2
Affects sports performance (‘*definitely* yes’)	51.0	53.5	48.8	58.3	59.2	57.4
Makes you gain/lose weight (‘*no difference’*)	42.3	37.7	46.5	42.6	45.2	40.1
Social influences						
Mother smoking	40.7	39.5	41.7	34.9	32.2	37.5
Father smoking	43.6	47.4	40.2	38.2	38.0	38.4
Sibling smoking	10.8	10.5	11.0	9.2	8.0	10.4
Friend smoking^a^	18.7	25.4	12.6	17.1	22.9	11.2

Notes: IMD, Indices of multiple deprivation score; ^a^at least one friend smokes or tried

Smoking behaviour data is reported for descriptive purposes. Self-reported smoking prevalence at T1 (comparison group, 97.5 %; intervention group, 98.6 %) and T2 (comparison group: 97.1 %, intervention group, 98.2 %) remained similar to T0, suggesting that low rates of smoking continued over time.

### Intervention effects

#### Non-smoking intentions

The adjusted multilevel linear regression analyses indicated no significant intervention effects on non-smoking intentions between baseline and T1 (Table 2), and between baseline and T2 (Table 3).

**Table 2:** Multilevel analyses of the effectiveness of the SmokeFree Sports intervention between baseline (T0) and post-intervention (T1)

	Mean difference^a^ (T1-T0: M ± SD or %)		Crude model^b^		Adjusted model^c^	
Outcome measure			β or RR (95 % CI)	P	β or RR (95 % CI)	P
Smoking intentions						
Total non-smoking intentions	I	0.03 ± 0.98	0.03^d^ (−0.07, 0.13)	0.51	0.02^d^ (−0.08, 0.12)	0.71
	C	0.07 ± 0.95				
Cigarette refusal self-efficacy						
Total refusal self-efficacy	I	0.29 ± 3.52	0.28^d^ (−0.10, 0.67)	0.15	0.28^d^ (−0.11, 0.67)	0.17
	C	0.15 ± 3.77				
Attitudes towards smoking						
Smoking is bad for health (‘*definitely yes’*)	I	3.8	1.03^e^ (0.99, 1.06)	0.19	1.03^e^ (0.99, 1.06)	0.15
	C	2.5				
Safe to smoke…year or two (‘*definitely not’*)	I	3.1	1.23^e^(1.11, 1.36)	<0.001*	1.19^e^ (1.07, 1.33)	0.01
	C	−5.0				
Difficult to quit once started (‘*definitely yes’*)	I	15.4	1.58^e^ (1.41, 1.78)	<0.001*	1.56^e^ (1.38, 1.76)	<0.001*
	C	−0.5				
Others smoke is harmful to you (‘*definitely yes’*)	I	2.4	1.19^e^ (1.07, 1.31)	<0.01*	1.19^e^ (1.20, 2.08)	<0.01*
	C	−2.5				
Affects sports performance *(‘definitely yes’)*	I	30.3	1.70^e^ (1.58, 1.85)	<0.001*	1.73^e^ (1.59, 1.88)	<0.001*
	C	0.9				
Makes you gain or lose weight (‘*no difference’*)	I	26.3	2.11^e^ (1.85, 2.41)	<0.001*	2.13^e^ (1.86, 2.44)	<0.001*
	C	−9.1				

Notes: β = beta coefficient; RR = relative risk; CI = confidence interval, I = intervention group; C = comparison. Values reflect the intervention effects (i.e., between-group differences) between baseline and post-intervention

*Significant intervention effect (*P* < 0.05)

^a^Unadjusted within-group mean difference (post-intervention minus baseline)

^b^Adjusted for group and baseline value of the outcome measure

^c^Additionally adjusted for school and deprivation level, sex, age, ethnicity, other individual smoking-related cognitions at baseline, enjoyment of physical activity and mother/father/sibling/friend smoking

^d^β value

^e^Relative risk

**Table 3:** Multilevel analyses of the effectiveness of the SmokeFree Sports intervention between baseline (T0) and one year follow-up (T2)

	Mean difference^a^ (T2-T0: M ± SD or %)		Crude model^b^		Adjusted model^c^	
Outcome measure			β or RR (95 % CI)	P	β or RR (95 % CI)	P
Smoking intentions						
Total non-smoking intentions	I	0.06 ± 0.97	0.08^d^ (−0.02,0.18)	0.13	0.08^d^ (−0.02,0.17)	0.14
	C	0.06 ± 1.18				
Cigarette refusal self-efficacy						
Total refusal self-efficacy	I	0.56 ± 3.23	0.27^d^ (−0.02,0.56)	0.07	0.23^d^ (−0.07,0.52)	0.13
	C	0.45 ± 3.73				
Attitudes towards smoking						
Smoking is bad for health (‘*definitely yes’*)	I	5.5	0.98^e^ (0.96,1.01)	0.28	0.99^e^ (0.97,1.01)	0.42
	C	8.3				
Safe to smoke…year or two (‘*definitely not’*)	I	−0.6	1.05^e^ (0.95,1.16)	0.42	1.03^e^ (0.93,1.15)	0.65
	C	0.4				
Difficult to quit once started (‘*definitely yes’*)	I	5.8	1.15^e^ (1.02,1.29)	0.05	1.15^e^ (1.02,1.3)	0.06
	C	6.6				
Others smoke is harmful to you (‘*definitely yes’*)	I	0.0	1.14^e^ (1.08,1.81)	0.03	1.13^e^ (1.05,1.79)	0.05
	C	−2.0				
Affects sports performance *(‘definitely yes’)*	I	23.2	1.55^e^ (1.42,1.69)	<0.001*	1.57^e^ (1.43,1.72)	<0.001*
	C	1.3				
Makes you gain or lose weight (‘*no difference’*)	I	−2.0	1.05^e^ (0.90,1.22)	0.60	0.98^e^ (0.83,1.16)	0.84
	C	−3.7				

Notes: β = beta coefficient; RR = relative risk; CI = confidence interval; I = intervention group; C = comparison. Values reflect the intervention effects (i.e., between-group differences) between baseline and post-intervention

*Significant intervention effect (*P* < 0.05)

^a^Unadjusted within-group mean difference (one year follow-up minus baseline)

^b^Adjusted for group and baseline value of the outcome measure

^c^Additionally adjusted for school and deprivation level, sex, age, ethnicity, other individual smoking-related cognitions at baseline, enjoyment of physical activity and mother/father/sibling/friend smoking

^d^β value

^e^Relative risk

#### Cigarette refusal self-efficacy

The adjusted multilevel linear regression analyses showed no significant intervention effects between baseline and T1 (Table 2), and between baseline and T2 (Table 3), for refusal self-efficacy.

#### Attitudes towards smoking

The adjusted multilevel logistic regression analyses indicated small positive intervention effects between baseline and T1 (Table 2) for four attitude towards smoking items. At T1, compared with children in the comparison group, children that participated in the SFS intervention were more likely to ‘definitely’ believe that: ‘it is not safe to smoke for a year or two as long as you quit after that’ (RR = 1.19, 95 % CI 1.07 to 1.33, *p* < 0.001), ‘it is difficult to quit smoking once started’ (RR = 1.56, 95 % 1.38 to 1.76, *p* < 0.001), ‘smoke from other peoples’ cigarettes is harmful to you’ (RR = 1.19, 95 % CI 1.20 to 2.08, *p* < 0.001), and ‘smoking affects sports performance’ (RR = 1.73, 95 % CI 1.59 to 1.88, *p* < 0.001). In addition, a moderate positive intervention effect was observed on the attitude item: ‘smoking makes ‘no difference’ to weight’ (RR = 2.13, 95 % CI 1.86 to 2.44, *p* < 0.001). Between baseline and T2 (Table 3), significant between-group differences remained on only one of the six attitude items. Compared with children in the comparison group, children who received the SFS intervention were more likely to ‘definitely’ believe that ‘smoking affects sports performance’ (RR = 1.57, 95 % CI, 1.43, 1.72, *p* < 0.001), a small positive intervention effect.

#### Sex interaction effects

Tables 4 and 5 show the results of the sex interaction analyses between baseline and T1, and between baseline and T2, respectively. Between baseline and T1, sex moderated the intervention effects for cigarette refusal self-efficacy; a significant small positive intervention effect was found in girls (β = 0.72, 95 % CI 0.21 to 1.23, *p* < 0.01) but not boys (β = -0.18, 95 % CI -0.77 to 0.41, *p* = 0.54). No other sex interactions were observed.

**Table 4:** Multilevel analyses exploring interaction effects by sex between baseline (T0) and post-intervention (T1)

		Mean difference^a^ (T1-T0: M ± SD or %)		Intervention* sex^b^ (crude model)		Boys^c^		Girls^c^	
Outcome measure		Boys	Girls	β or RR (95 % CI)	P	β or RR (95 % CI)	P	β or RR (95 % CI)	P
Smoking intentions									
Total non-smoking intentions	I	0.10 ± 1.2	−0.04 ± 0.7	0.10^d^ (−0.10, 0.30)	0.32	n/a	−	n/a	-
	C	0.16 ± 1.0	0.00 ± 0.9						
Cigarette refusal self-efficacy									
Total refusal self-efficacy	I	0.32 ± 3.9	0.25 ± 3.2	0.87^d^ (0.09, 1.64)	0.03*	−0.18^d^ (−0.77, 0.41)	0.54	0.72^d^ (0.21, 1.23)	<0.01*
	C	0.39 ± 3.6	−0.07 ± 3.8						
Attitudes towards smoking									
Smoking is bad for health (‘*definitely yes’*)	I	5.5	2.0	1.05^e^ (1.00, 1.11)	0.12	n/a	-	n/a	-
	C	4.4	0.8						
Safe to smoke…year or two (‘*definitely not’*)	I	0.9	5.3	1.16^e^ (0.95, 1.42)	0.23	n/a	-	n/a	-
	C	−4.4	−5.6						
Difficult to quit once started (‘*definitely yes’*)	I	17.4	13.5	1.19^e^ (0.90, 1.57)	0.30	n/a	-	n/a	-
	C	2.6	−3.1						
Others smoke harmful to you (‘*definitely yes’*)	I	3.3	1.4	1.14^e^ (0.84, 2.23)	0.29	n/a	-	n/a	-
	C	−0.9	−4.0						
Affects sports performance *(‘definitely yes’)*	I	30.3	30.3	1.16^e^ (0.90, 1.50)	0.34	n/a	-	n/a	-
	C	5.3	−3.1						
Makes you gain or lose weight (‘*no difference’*)	I	23.4	29.1	1.21^e^ (0.79, 2.31)	0.36	n/a	-	n/a	-
	C	−2.6	−15.0						

Notes: β = beta coefficient; RR = relative risk; CI = confidence interval; I = intervention group; C = comparison. Where crude analyses were significant, adjusted analyses (i.e., interaction term included in adjusted multilevel models) were conducted with results for each sex shown

^*^Significant intervention effect (*P* < 0.05)

^a^Unadjusted within-group mean difference (post-intervention minus baseline)

^b^Adjusted for group and baseline value of the outcome measure

^c^Additionally adjusted for school and deprivation level, sex, age, ethnicity, other individual smoking-related cognitions at baseline, enjoyment of physical activity and mother/father/sibling/friend smoking

^d^β value

^e^Relative risk

**Table 5:** Multilevel analyses exploring interaction effects by sex between baseline (T0) and one year follow-up (T2)

		Mean difference^a^ (T2-T0: M ± SD or %)		Intervention* sex^b^ (crude model)		Boys^c^		Girls^c^	
Outcome measure		Boys	Girls	β or RR (95 % CI)	P	β or RR (95 % CI)	P	β or RR (95 % CI)	P
Smoking intentions									
Total non-smoking intentions	I	0.10 ± 1.2	0.00 ± 0.7	−0.01^d^ (−0.21, 0.19)	0.89	n/a	-	n/a	-
	C	0.06 ± 1.4	0.06 ± 0.9						
Cigarette refusal self-efficacy									
Total refusal self-efficacy	I	0.84 ± 3.4	0.27 ± 3.0	−0.20^d^(−0.78, 0.39)	0.51	n/a	-	n/a	-
	C	0.37 ± 3.8	0.53 ± 3.7						
Attitudes towards smoking									
Smoking is bad for health (‘*definitely yes’*)	I	8.3	2.5	1.02^e^ (1.00, 1.04)	0.17	n/a	-	n/a	-
	C	10.5	6.3						
Safe to smoke…year or two (‘*definitely not’*)	I	−0.5	−0.9	0.94^e^ (0.76, 1.17)	0.64	n/a	-	n/a	-
	C	−2.6	3.1						
Difficult to quit once started (‘*definitely yes’*)	I	7.8	4.0	1.03^e^ (0.81, 1.31)	0.84	n/a	-	n/a	-
	C	6.2	7.1						
Others smoke harmful to you (‘*definitely yes’*)	I	1.6	−1.7	1.11^e^ (0.77, 2.15)	0.41	n/a	-	n/a	-
	C	−0.9	−3.2						
Affects sports performance *(‘definitely yes’)*	I	25.1	21.3	1.05^e^ (0.83, 1.32)	0.73	n/a	-	n/a	-
	C	5.3	−2.3						
Makes you gain or lose weight (‘*no difference’*)	I	−2.5	−1.7	0.99^e^ (0.26, 3.70)	0.99	n/a	-	n/a	-
	C	1.8	−8.7						

Notes: β = beta coefficient; RR = relative risk; CI = confidence interval; I = intervention group; C = comparison. Where crude analyses were significant, adjusted analyses (i.e., interaction term included in adjusted multilevel models) were conducted with results for each sex shown

^a^Unadjusted within-group mean difference (one year follow-up minus baseline)

^b^Adjusted for group and baseline value of the outcome measure

^c^Additionally adjusted for school and deprivation level, sex, age, ethnicity, other individual smoking-related cognitions at baseline, enjoyment of physical activity and mother/father/sibling/friend smoking

^d^β value

^e^Relative risk

#### Qualitative findings

During focus groups the majority of children articulated that SFS made them more determined not to smoke in the future. Children’s reasons for not smoking surrounded some of the key messages received and or activities played during the intervention (see Table 6 for a summary of children’s reasons for their non-smoking intentions). During group discussions some children expressed a level of uncertainty regarding their future smoking behaviour and verbalised that they may smoke in the future because of social norms, and or using smoking as a coping mechanism for managing stress:

“*I don’t want to smoke when I’m older but I’ll probably end up changing my mind because I want to be like one of my sisters who smokes” (Girl, School 1).*

“*I’m not saying I definitely won’t [smoke] because it’s just something that might happen if something stressful happens” (Boy, School 2).*

**Table 6:** Children’s reasons for their non-smoking intentions

Health messages	Quotes
*Health implications of smoking*	*“I won’t [smoke] because if you smoke you will damage your heart and if you don’t smoke you can live a long and healthy life” (Girl, School 7).* *“There’s a reason why I wouldn't smoke because your lungs wouldn't be in the best condition” (Boy, School 10).*
*Impact on sports performance*	*“I wouldn’t [smoke] because It’s harder to breathe and harder to do exercise” (Boy, School 9).* *“Because I like doing lots of sports and if I smoked in the future it would be difficult to do [sports]” (Boy, School 8)*.
*Cigarette contents and addiction*	*“Because I’ve learnt [in SFS] how many chemicals are in a cigarette and what goes into them, that’s why I wouldn’t smoke” (Boy, School 8).* *I’m very confident that I’m not going to smoke cos they’ve [SFS] told us how bad it [smoking] is and there are over four thousand chemicals [in a cigarette] and it can be hard to quit” (Girl, School 1).*
*Financial costs of smoking*	*“I wouldn’t [smoke] because it costs you loads of money”(Girl, School 5)*.

During group discussions children were able to recall the health messages delivered during the intervention, particularly in relation to the health implications associated with smoking, its impact on sport performance, the chemical properties in a cigarette and its addictive nature, and its impact on weight (see Table 7).

**Table 7:** Children’s’ understanding of the health messages received during the intervention

Health messages	Quotes
*Health implications of smoking*	*“The [SFS] games show you the damage that [smoking] does to your arteries and lungs” (Boy, School 3).* *“A smoker would get more phlegm and a non-smoker would get less phlegm” (Boy, School 4, Gp 1).*
*Impact on sports performance*	*“Your heart beats faster when you’re doing exercise when you’re a smoker” (Boy, School 5).* *“If you smoked you wouldn’t be able to run as long or play as long as other [non-smoking] people” (Boy, School 3).*
*Cigarette contents and addiction*	*“There’s over 4,000 chemicals in a cigarette and they’re not nice, rat poison, nicotine, rocket fuel” (Girl, School 6).* *“I wouldn’t [smoke] because it’s addictive and you won’t be able to stop cos of the nicotine” (Girl, School 5).*
*Impact on weight*	*“People think when you smoke you lose weight but you really don’t” (Boy, School 5).* *“It’s healthier not to smoke and there’s no difference in your weight, you’re just better off not smoking”(Boy, School 6).*

## Discussion

This study examined the short and medium term effects of a sport-for-health intervention (SFS) on 9-10 year old children’s intentions (not) to smoke and smoking-related cognitions (attitudes and refusal self-efficacy) using a controlled trial. The SFS intervention had no effect on children’s ratings of non-smoking intentions, though qualitative data suggested that participation in SFS made children more determined not to smoke. In addition, a small positive short term effect was found for refusal self-efficacy among girls in the intervention group. Participation in SFS also increased the likelihood of having negative attitudes towards smoking immediately after the intervention, with children stating that the intervention reinforced non-smoking opinions, though limited effects were found one year after the intervention.

Smoking intentions are precursors to and predictive of smoking initiation in youth [8, 68]. Quantitative data indicated that the SFS intervention did not significantly impact on children’s smoking intentions; children in both the intervention and comparison group reported a strong intention not to smoke in the future and thus a ceiling effect limited our ability to detect between-group differences. These findings are comparable to other smoking prevention programmes targeting primary school aged children [19, 69], but are inconsistent with two school-based interventions that reported a positive effect on intentions to smoke among elementary school children from the USA [70–72]. A further study examined the immediate and long term effects of a smoking education programme implemented in Dutch elementary schools [73]. The study reported no short term effects on intention to smoke during elementary school. However, when children were followed up at secondary school one year after the intervention, children who received the intervention had significantly higher non-smoking intentions and smoked less than the control group [73]. The above mentioned studies might suggest that smoking prevention interventions may be more effective if implemented in secondary school, as children may be more likely to have developed intentions to smoke if they are closer to the actual age of smoking onset (i.e., age 14-16 years [19, 73]. Nevertheless, given that qualitative data suggested that SFS had strengthened children’s non-smoking intentions, a longer term follow-up study is warranted to investigate whether implementing SFS during primary school is effective at reducing smoking behaviour and smoking intentions in adolescence, following the transition to secondary school.

Children’s intentions to smoke can be shaped by their attitudes towards smoking and their self-efficacy expectations [9, 10, 74, 75]. Smoking-related knowledge and attitudes are frequently measured and have a propensity to increase following smoking prevention interventions [72, 76–78]. Consistent with these studies, children who participated in the SFS intervention were more likely to develop negative attitudes towards smoking immediately following the intervention than children in the comparison group. Focus group data with children supported these findings and revealed that the SFS games and smoke free messages positively influenced children. Given that many preadolescent children living in socially deprived communities display pro-smoking attitudes [14], these findings are encouraging and suggest that SFS could therefore provide a mechanism for health education to dispel myths that exist among children around smoking harms and challenges. However, it is also worth noting that the majority of the positive intervention effects on attitudes had diminished one year after the intervention. Though not directly comparable due to methodological differences, these results are in accord with Crone and colleagues [72], who also noted a number of short term positive between-group effects on attitudes towards smoking that had reduced by long term follow-up. It is possible, therefore, that additional ‘booster’ sessions may be necessary to sustain attitude changes in preadolescent children, particularly those residing in deprived communities. However, the evidence on the effectiveness of booster sessions is limited and inconsistent [79].

The likelihood of starting to smoke increases in adolescence [80, 81] and so enhancing skills to resist social pressures to smoke is important for smoking prevention [82]. Whilst a small positive effect was observed on cigarette refusal self-efficacy among girls in the intervention group at post-intervention, no group differences were apparent at one-year follow-up. It is possible that short term intervention effects on refusal self-efficacy were not found in boys because efficacy levels increased from baseline to post-intervention among boys in both the intervention and comparison groups. It is also possible that short term effects on girls’ refusal self-efficacy were not maintained at one-year follow-up because girls’ in the comparison groups ratings increased and they appeared to ‘catch up’. These mixed findings likely reflect that self-efficacy is not a static concept [83] and levels of self-efficacy fluctuate over time [80]. The findings are similar to those reported by Isensee et al. [78], who also noted a lack of medium term effects and documented increases in refusal self-efficacy among control group participants. Further, the absence of intervention effect could again be attributed to a ceiling effect; children in the intervention and comparison groups both reported high refusal self-efficacy, reducing the power to detect noticeable effects. It is also worth noting that most children in the current study did not have friends who smoke, and so have yet to be put to the test of resisting social influences to smoke. Given that self-efficacy is subject to change over time, it has been recommended that smoking prevention programmes are implemented annually in preadolescence and throughout adolescence until the completion of secondary school [35, 77, 80]. Long-term research is required to determine if the SFS primary school smoking prevention intervention can facilitate children in making a rational and logical decision not to smoke during a period when smoking is more age-related and considered as accepted behaviour [84].

To the authors knowledge SFS was the first sport-for-health intervention to engage children in smoking prevention. A recently published process evaluation of the intervention suggests that this unique approach was well-received by children, and was considered acceptable to coaches and teachers as intervention deliverers [64]. However, there were variations in intervention fidelity and teachers’ implementation of intervention activities that may have reduced the potency of the intervention and the ability to sustain short term effects one year after the intervention [64]. Nevertheless, the limited intervention effects are more likely attributed to children at this age having strong intentions not smoke in the future, though important lessons have been learned that can inform the design of a randomised controlled trial [64].

The present study has several strengths. First, in accordance with MRC guidance for the development of complex interventions, SFS was designed following extensive formative work, school and community feasibility studies [29, 41, 42]. Second, this study adopted a mixed-methodology approach consistency with the Standard Evaluation Framework for physical activity interventions [85]. Third, this study followed children one year after the end of the intervention. Fourth, the study had a large sample size and reasonably low attrition rates were observed. Fifth, process evaluation measures were used to explore the implementation of SFS [64]. Finally, to the date, the results of this study provide the first globally published evidence for the effectiveness of a large scale school-based sport-for-health smoking prevention intervention.

Several limitations should be acknowledged. First, of the 154 schools approached, only 43 agreed to participate. Previous research has shown that some parents and school officials may be concerned that exposing preadolescent children to smoking prevention programmes may stimulate their interest and curiosity about smoking [86]. However, the primary reason given by schools for non-participation was limited time, which might be related to the fact that smoking prevention is not mandatory in Key Stage 2 of the UK National Curriculum [53]. Further, it was encouraging to note that participation in SFS did not increase rates of smoking initiation. Second, the reliance on self-report in the assessment of outcome variables carries a risk of measurement error due to inaccurate recall, literacy issues and social desirability bias [82, 87]. However, self-reports have been demonstrated to be accurate provided confidentially is assured [88]. Third, it was not possible to blind study participants or the research team to the intervention because of the practical nature of the intervention. Fourth, primary and secondary outcomes focused on intentions to smoke and smoking-related cognitions, respectively, which may or may not result in smoking initiation at a later age [13]. Fifth, given that the majority of children were White British and from one of the most deprived local authorities in England, these results may not generalise to other racial and socio-economic child populations. Sixth, focus groups were only conducted with children from intervention schools to inform interpretations of intervention effectiveness and therefore it is unknown if children from comparison schools would have conveyed similar perspectives. Finally, the study did not include a cost-effectiveness evaluation, thus it is unknown whether SFS is a cost-effective smoking prevention initiative.

## Conclusions

In summary, the results of this study indicate that SFS was effective at changing attitudes towards smoking, and increasing the level of individual self-efficacy to refuse cigarettes among girls immediately post-intervention. Although no quantitative intervention effects were observed for non-smoking intentions, children articulated that SFS made them more determined not to smoke. Overall, these findings may suggest that sport-for-health interventions offer a promising strategy for smoking prevention efforts, though a long term follow-up study is needed to determine whether the SFS intervention is effective at preventing smoking in secondary school. Moreover, further evidence is needed from randomised controlled-trials. The fact that almost all children had not developed an intention to smoke might indicate that smoking prevention programmes should target early adolescents (aged 11-13 years), who are closer to the actual age of smoking onset.
